# Drug-Induced Subacute Cutaneous Lupus Secondary to Sulfasalazine

**DOI:** 10.7759/cureus.105393

**Published:** 2026-03-17

**Authors:** Kristal Choi, Linh Truong, Yuna Kang

**Affiliations:** 1 Division of Rheumatology, Department of Medicine, University of California, Los Angeles, David Geffen School of Medicine, Los Angeles, USA; 2 Department of Pathology and Laboratory Medicine, University of California, Los Angeles, David Geffen School of Medicine, Los Angeles, USA

**Keywords:** drug-induced lupus, drug-related side effects and adverse reactions, photosensitivity disorder, subacute cutaneous lupus erythematosus, sulfasalazine

## Abstract

This is a rare case of a patient with rheumatoid arthritis and Sjögren’s disease who developed drug-induced subacute cutaneous lupus erythematosus (DI-SCLE) while on sulfasalazine. Diagnosis was made by skin biopsy and confirmed by improvement of the rash with cessation of the offending medication. Sulfasalazine is implicated in drug-induced lupus erythematosus (DILE) but less so in DI-SCLE, although this may be in part due to underreporting from lack of biopsy confirmation and diagnostic uncertainty caused by the overlap in systemic and cutaneous lupus presentations. Drug-induced photosensitivity while on sulfasalazine and Sjögren syndrome-related antigen A (SSA) antibody positivity may have predisposed the patient to develop DI-SCLE in this case. It is always important to maintain a level of concern for drug-related adverse effects in a patient presenting with subacute cutaneous lupus erythematosus (SCLE) so that the offending drug can be promptly discontinued.

## Introduction

Subacute cutaneous lupus erythematosus (SCLE) is a subset of cutaneous lupus erythematosus, typically characterized by non-scarring annular or papulosquamous eruptions on sun-exposed skin. Patients rarely develop serious systemic involvement [1]. Drug-induced SCLE (DI-SCLE) was first described in 1985 by Reed et al., who noted five patients who developed classic SCLE rashes while on hydrochlorothiazide. Notably, all patients were positive for Sjögren syndrome-related antigen A (SSA) autoantibodies [2]. Since then, many other medications have been implicated in DI-SCLE, and these include calcium channel blockers, terbinafine, angiotensin-converting enzyme inhibitors, and anti-tumor necrosis factor agents [3,4].

Sulfasalazine is a disease-modifying anti-rheumatic drug (DMARD) that is used as a common treatment in many rheumatic diseases, including rheumatoid arthritis. Reports exist of patients developing drug-induced lupus erythematosus (DILE) while on sulfasalazine. In these cases, the clinical presentation is characterized by serositis, arthralgias, constitutional symptoms, and positive autoantibodies (antinuclear antibody, double-stranded deoxyribonucleic acid antibody, histone antibody) [5-10]. Reports of sulfasalazine causing DI-SCLE specifically are not well-documented in the literature.

Here, we present a case of DI-SCLE in a patient with seropositive rheumatoid arthritis and Sjögren’s disease and highlight the key serologic and clinical features that distinguish this from classic DILE.

## Case presentation

We present a case of a 70-year-old Caucasian female with a known history of seropositive rheumatoid arthritis and Sjögren’s disease. The patient had a long-standing history of rheumatoid arthritis, for which she had been on monotherapy with sulfasalazine 1000 mg twice daily. Notably, she had been treated with both hydroxychloroquine and methotrexate in the past, both of which caused adverse effects requiring discontinuation.

**Figure 1:**
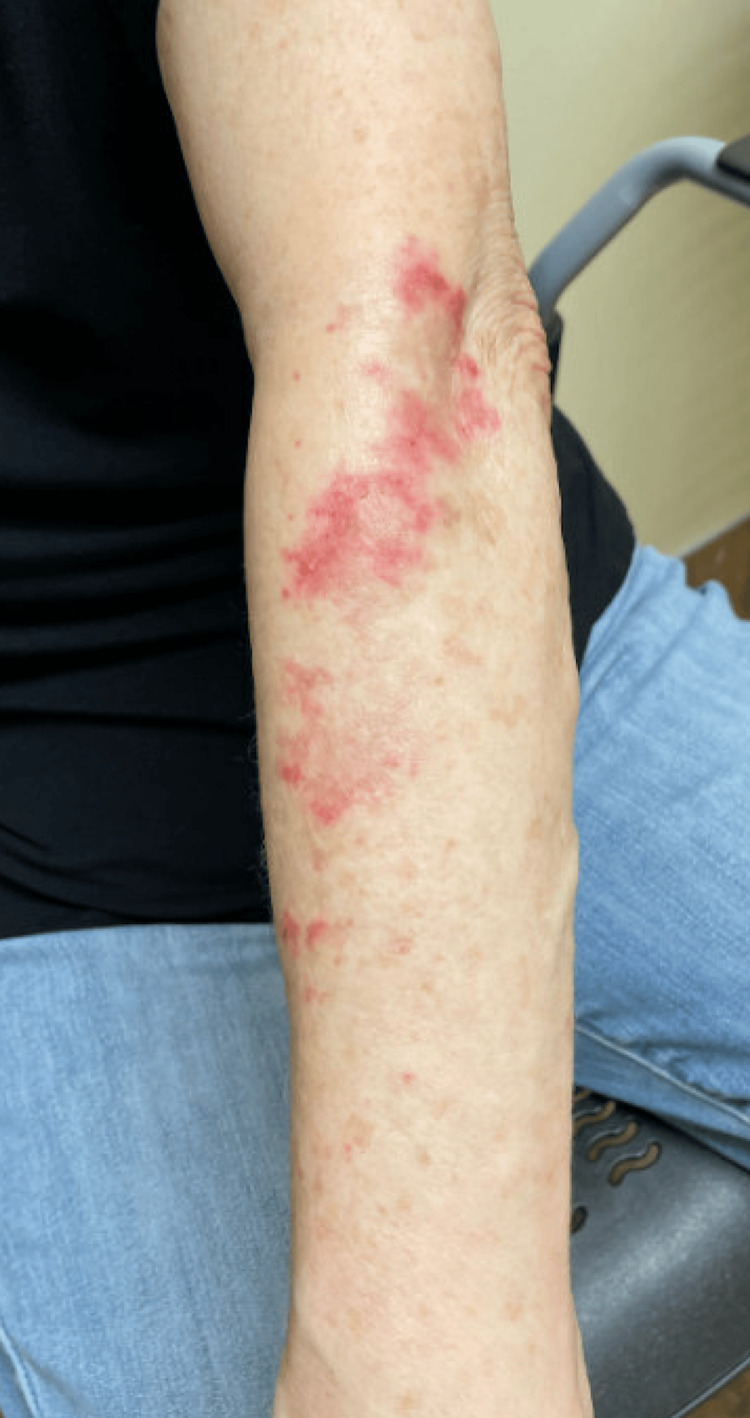
Erythematous annular rash on the left upper extremity.

During one of her routine follow-up visits, the patient reported a two-month history of a new onset of a pruritic rash consisting of erythematous, annular plaques with central clearing that coalesced into polycyclic patches across the photodistributed bilateral upper extremities (Figure 1). She denied any other new constitutional, mucocutaneous, serosal, or musculoskeletal symptoms. At that time, the patient had been on sulfasalazine for approximately three years. The patient’s antibody profile was obtained, and pertinent positives included positive SSA and cyclic citrullinated (CCP) antibodies with notably negative antinuclear antibody (ANA) and histone antibody (Table 1).

**Table 1:** Pertinent lab results at baseline. Ab, antibody; ANA, antinuclear antibody; CCP, cyclic citrullinated peptide; CRP, C-reactive protein; DRVVT, dilute Russell viper venom time; dsDNA, double-stranded deoxyribonucleic acid; EIA, enzyme immunoassay; ESR, erythrocyte sedimentation rate; HPF, high-power field; IFA, indirect fluorescent antibody; nDNA, native deoxyribonucleic acid; RF, rheumatoid factor; RNP, ribonucleoprotein; SM, Smith antigen; SSA, Sjögren syndrome-related antigen A (Ro); SSB, Sjögren syndrome-related antigen B (La).

Pertinent lab data	Patient’s lab values	Reference range
ANA (titer)	<1:40	<1:40
nDNA (Crithidia) Ab IFA (titer)	<1:10	<1:10
dsDNA EIA (IU/mL)	<=200	<=200
SM Ab (U)	<20	<20
RNP Ab (U)	<20	<20
SSA Ab (U)	66	<20
SSB Ab (U)	<20	<20
Cardiolipin IgG (CU)	<20	<20
Cardiolipin IgA (CU)	<20	<20
Cardiolipin IgM (CU)	<20	<20
Beta-2-glycoprotein IgG (SGU)	<10	<10
Beta-2-glycoprotein IgA (SMU)	<10	<10
Beta-2-glycoprotein IgM (SAU)	<10	<10
DRVVT interpretation	Negative	Negative
RF (IU/mL)	<10	<10
CCP IgG (Units)	>250	0-19
ESR (mm/hr)	9	<=25
CRP (mg/dL)	0.3	<0.8
C3 (mg/dL)	123	86-176
C4 (mg/dL)	22	10-40
Histone antibody, IgG (Units)	0.3	0.0-0.9
Protein/creatinine ratio, urine	0.3	0.0-0.4
RBC per HPF, urine	0	0-2

**Figure 2:**
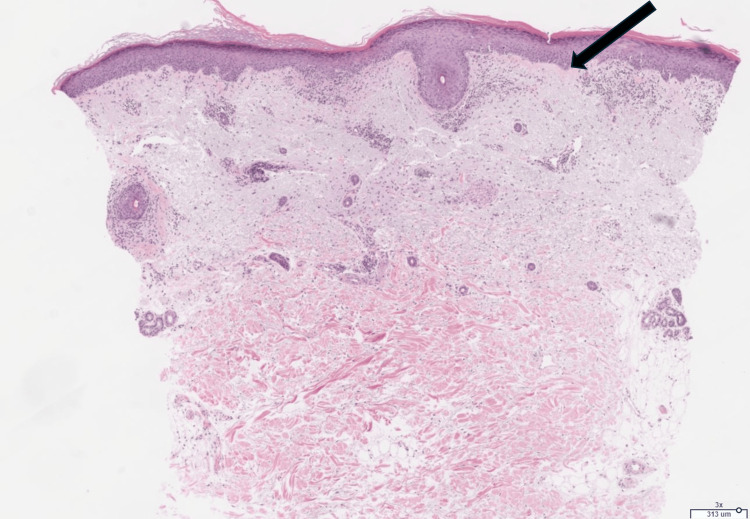
H&E (low-power, 3x). Arrow denotes interface dermatitis with predominantly superficial dermal, perivascular, and periadnexal lymphocyte predominant inflammation.

**Figure 3:**
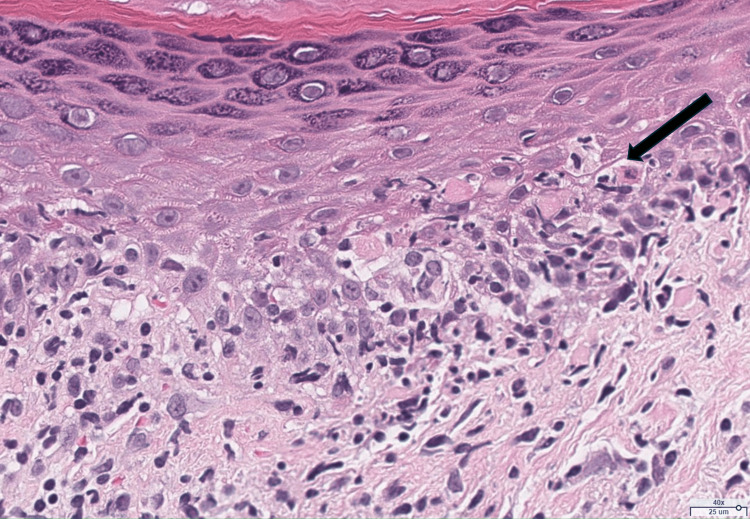
H&E (high-power, 40x). Arrow denotes epidermal interface dermatitis with vacuolar interface changes in the basal layer and necrotic keratinocytes.

**Figure 4:**
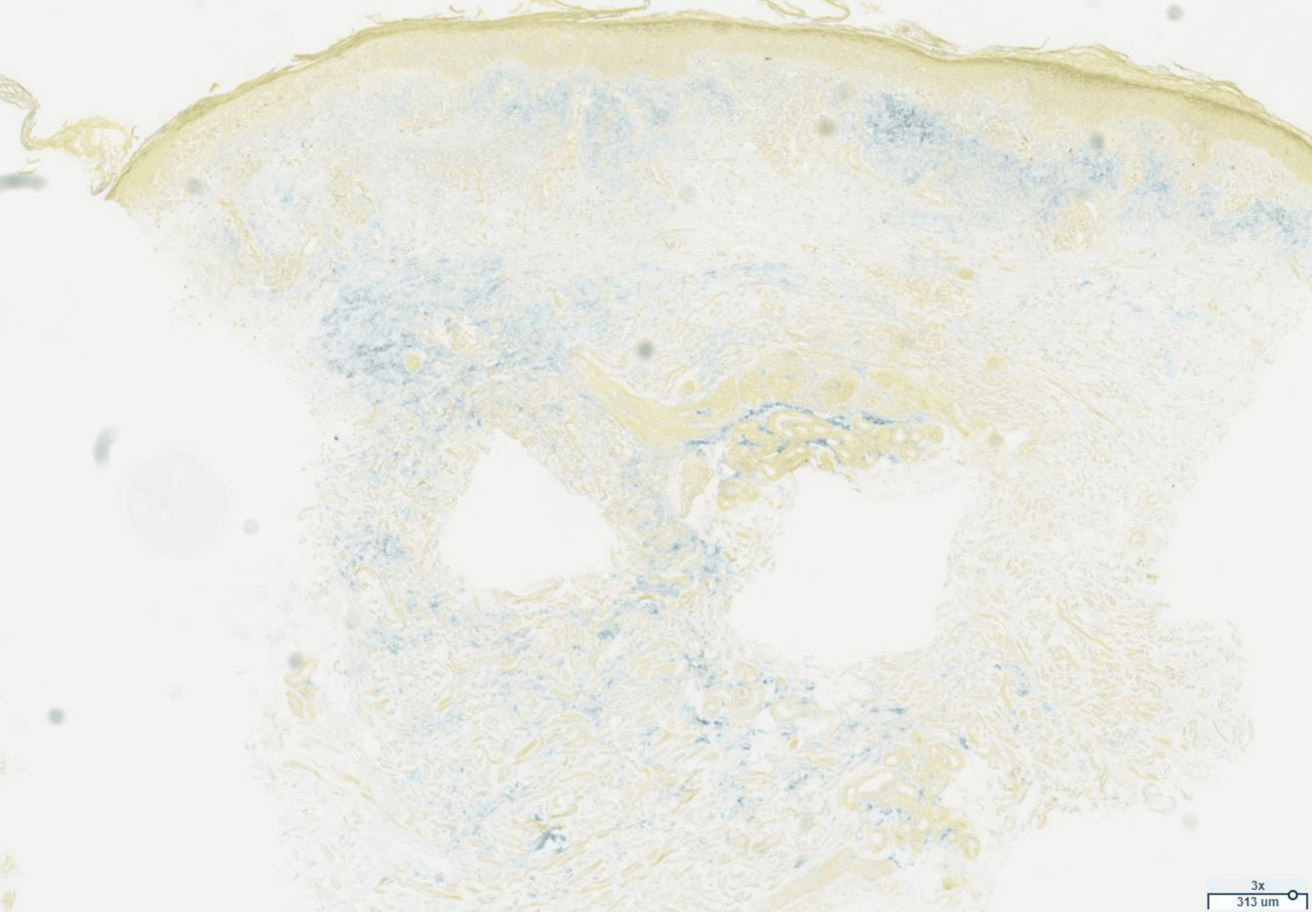
Colloidal iron stain (low-power, 3x). Increased dermal mucin (appears blue).

She was seen by dermatology one month later. Differential diagnosis included SCLE, tinea corporis, and erythema annulare centrifugum. Skin biopsy was performed on the same visit, which showed the following: interface dermatitis with superficial/deep dermal perivascular and periadnexal lymphoplasmacytic inflammation, and increased dermal mucin seen on alcian blue and colloidal iron stains (Figures 2-4). Based on the clinical features and histopathology of the rash, the patient was diagnosed with SCLE. The patient was offered additional treatment for SCLE, including but not limited to a re-trial of hydroxychloroquine or methotrexate, but she declined these systemic medications. She was ultimately prescribed topical steroids, which helped partially with her symptoms.

**Figure 5:**
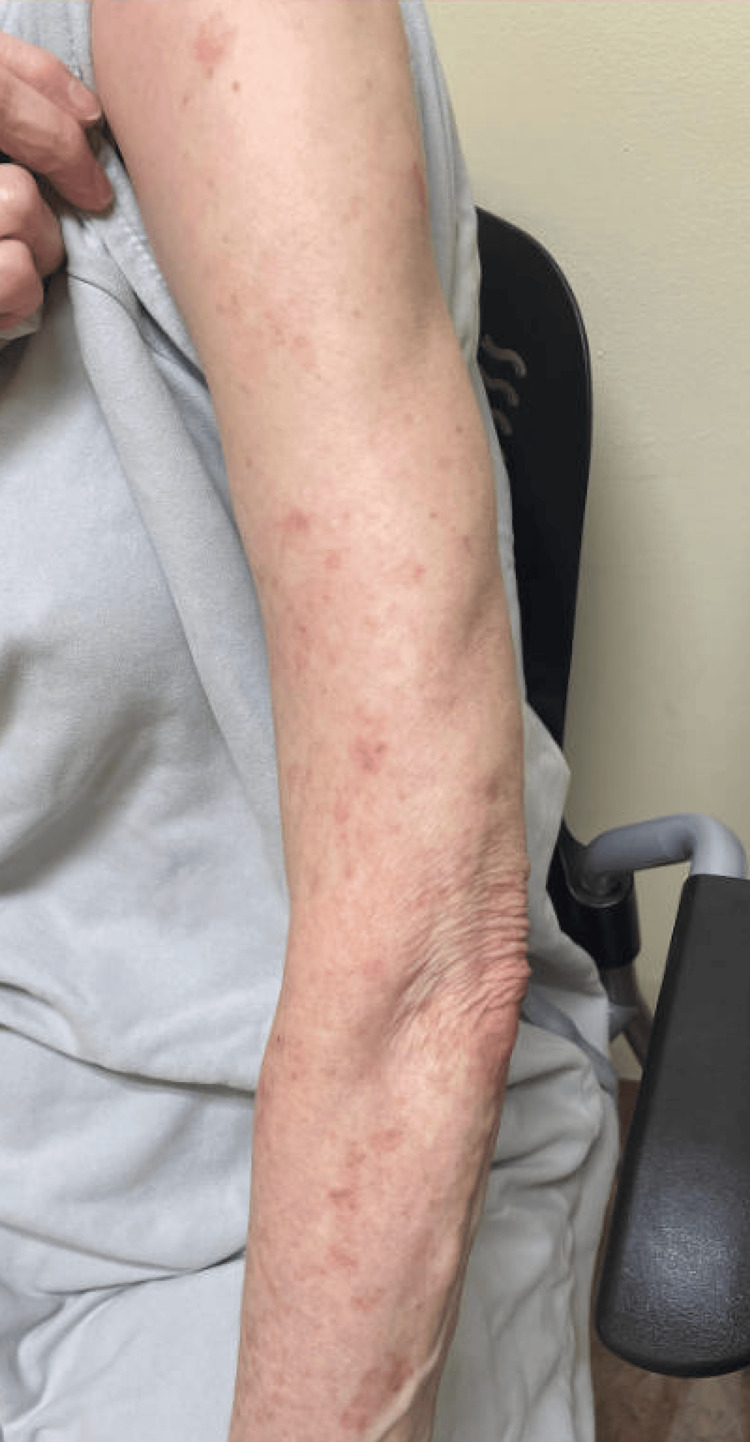
Improvement of the rash on the left upper extremity one year later.

Six months later, the patient developed an upper respiratory infection, during which time she was advised to temporarily stop sulfasalazine. During this time, the patient reported that her rash improved significantly. Given her clinical improvement, it was advised that she refrain from sulfasalazine, and her skin cleared substantially. The rash remained significantly improved one year later (Figure 5). The correlation raised concern for DI-SCLE, and sulfasalazine was permanently discontinued.

## Discussion

While sulfasalazine-induced DILE is well-documented [5-10], with reported cases typically demonstrating positive ANA, double-stranded deoxyribonucleic acid (dsDNA), and antihistone antibodies accompanied by serositis and constitutional symptoms, sulfasalazine-induced SCLE appears to be considerably rarer. Our case aligns with the characteristic serologic profile of DI-SCLE: positive SSA antibody with notably negative ANA, dsDNA, and histone antibodies, a pattern that distinguishes this entity from classic DILE.

Conflicting data exist regarding the incidence of DILE in patients on sulfasalazine: a prospective randomized trial of 200 rheumatoid arthritis patients treated with sulfasalazine or auranofin found that while 19% of initially ANA-negative sulfasalazine patients became strongly ANA-positive during treatment, no cases of drug-induced lupus were identified over the study period [11]. Risk factors for lupus-related side effects include human leukocyte antigen (HLA) DR 0301 haplotype, elevated serum IL-10 levels, and pre-existing antinuclear antibodies in speckled patterns [7], suggesting that immunomodulation associated with sulfasalazine treatment may contribute to lupus-related reactions in genetically predisposed individuals.

DI-SCLE is not thought to differ clinically, histopathologically, or immunologically from idiopathic SCLE [12]. While the exact etiology of DI-SCLE is not known, drug-induced photosensitivity may contribute, given that some of the implicated drugs are known to cause phototoxicity [4]. Notably, photosensitivity is a well-documented adverse effect of sulfasalazine, which contains a sulfonamide component that can become photo-activated when it absorbs ultraviolet [13]. A pre-existing predisposition is also likely, given that most reported cases involve patients with known SSA antibody positivity, as in our patient. Management of DI-SCLE involves immediate removal of the offending agent as well as treatments that are also used in SCLE (e.g., topical steroids and hydroxychloroquine) [1,4].

We did not find any well-documented case report or series in the literature that describes sulfasalazine causing DI-SCLE. However, DI-SCLE may be underrecognized due to overlap with systemic DILE and underreporting when sulfasalazine-associated rashes are not biopsied.

Sulfasalazine causes a spectrum of cutaneous rashes, ranging from mild rashes to rare, life-threatening presentations. According to the United States Food and Drug Administration (FDA), non-specific rashes occurred in 13% of rheumatoid arthritis patients on this medication. Severe cutaneous adverse reactions were rare but included Stevens-Johnson syndrome (SJS) and toxic epidermal necrosis (TEN), drug reaction with eosinophilia and systemic symptoms (DRESS), exfoliative dermatitis, acute generalized exanthematous pustulosis (AGEP), and erythema multiforme [14].

In patients with autoimmune diseases like inflammatory bowel disease and rheumatoid arthritis, distinguishing drug-related adverse effects from manifestations of underlying disease can be challenging. The temporal relationship between drug initiation and symptom onset can be helpful in this regard [15,16]. In our case, the long latency period between the initiation of the medication and the development of DI-SCLE delayed diagnosis, as sulfasalazine was not immediately implicated. Overall, there is considerable variability in the timeline for drug-induced lupus; one database found a median delay of 172 days between treatment initiation and lupus occurrence across all implicated drugs [17].

Several limitations warrant acknowledgment. First, the single-patient design precludes generalizability and causal inference. Second, serial serologic monitoring following sulfasalazine discontinuation was not performed, which could have provided additional evidence of immunologic resolution corresponding with clinical improvement. Third, rechallenge with sulfasalazine was not attempted, given the availability of alternative therapies and the potential for recurrent cutaneous disease.

## Conclusions

This case illustrates the development of biopsy-proven SCLE in a patient on sulfasalazine for treatment of rheumatoid arthritis. The subsequent improvement of the rash upon cessation of sulfasalazine confirmed that this was likely DI-SCLE in a patient who was genetically predisposed with known SSA positivity. The case further highlights the importance of maintaining a high index of suspicion for DI-SCLE in any patient presenting with a consistent rash, especially if the patient has positive SSA, so that the offending medication can be discontinued.

As with all single-case reports, the findings presented here cannot establish causality and may not be generalizable to all patients receiving sulfasalazine. Individual patient factors, including underlying autoimmune predisposition and genetic susceptibility, may influence the development of DI-SCLE and limit the applicability of these observations to broader populations.
